# Contrast-Induced Encephalopathy Due to Contrast Extravasation From a Chronic Post-hemorrhagic Encephalomalacic Region: Prior Hemorrhage as a Potential Risk Factor

**DOI:** 10.7759/cureus.105391

**Published:** 2026-03-17

**Authors:** Zhuo Luan, Seunghong Rhee, Mohammad Ashkar, Thomas O’Neill, Salvador Cruz-Flores

**Affiliations:** 1 Neurology, Texas Tech University Health Sciences Center, El Paso, USA; 2 Radiology, Texas Tech University Health Sciences Center, El Paso, USA; 3 Neuroradiology, Texas Tech University Health Sciences Center, El Paso, USA

**Keywords:** brain hemorrhage, contrast-induced encephalopathy, end stage renal disease (esrd), risk factor investigation, stroke

## Abstract

Contrast-induced encephalopathy (CIE) is a rare complication of iodinated contrast administration, typically presenting with transient neurological symptoms ranging from confusion to coma. It is most commonly reported after intra-arterial procedures involving large contrast volumes and occurs more frequently in patients with risk factors such as renal impairment, hypertension, and prior central nervous system (CNS) injury. We report a unique case of a 64-year-old woman with end-stage renal disease (ESRD) on hemodialysis, a remote right temporoparietal lobar hemorrhage, and a recent left thalamic hemorrhage who developed decreased consciousness following CT angiography (CTA) with a relatively low dose of intravenous contrast. Imaging revealed contrast extravasation localized to the site of her prior lobar hemorrhage, without evidence of new bleeding. The patient gradually improved with supportive care, including hemodialysis. To our knowledge, this is the first reported case of CIE caused by contrast leakage into a chronic post-hemorrhagic encephalomalacic region. This case suggests that longstanding blood-brain barrier (BBB) disruption from prior hemorrhage may predispose ESRD patients to contrast-induced neurotoxicity even at low contrast doses. Clinicians should exercise caution when administering contrast to patients with ESRD and prior CNS injury, and further studies are warranted to guide contrast use and preventive strategies in this high-risk population.

## Introduction

Contrast-induced encephalopathy (CIE) is a rare and often reversible complication resulting from the administration of iodinated contrast media. It presents with a broad clinical spectrum ranging from transient confusion and agitation to seizures, visual disturbances, cortical blindness, aphasia, hemiparesis, and even coma. The damage could be temporary or permanent. The incidence of CIE is estimated to be 0.3-4% [1]. Most reported cases of CIE occur after intra-arterial administration of large volumes of iodinated contrast, particularly during cerebral or coronary angiography. However, CIE is not exclusively associated with high-dose exposures; many cases have also been documented following low doses of contrast [2]. This underscores the importance of identifying individual risk factors, as even standard diagnostic contrast loads may precipitate encephalopathy in vulnerable patients. Nonetheless, the risk factors for CIE are not yet fully understood and remain an area of ongoing investigation.

The pathophysiology of CIE is not fully understood but is believed to involve blood-brain barrier (BBB) disruption, allowing contrast agents to penetrate the cerebral parenchyma. Once inside the brain, the contrast exerts a toxic effect on neurons and glial cells. Risk factors that predispose patients to CIE so far identified include advanced age, renal insufficiency, hypertension, diabetes mellitus, and ischemic stroke [3]. Patients with end-stage renal disease (ESRD) are particularly vulnerable to CIE due to impaired renal clearance of iodinated contrast agents, resulting in prolonged systemic exposure and delayed elimination [4]. Comorbid conditions such as advanced age, hypertension, and diabetes mellitus can contribute to microvascular disease and compromise the integrity of the BBB. Similarly, a history of ischemic stroke may cause focal disruption of the BBB in the affected or adjacent vascular territories [1,3,5]. These factors increase the likelihood of contrast leakage into previously injured brain tissue, potentially triggering localized or diffuse cerebral edema and neurotoxicity. Therefore, ESRD patients with a history of hypertension, diabetes, or ischemic stroke may be at even higher risk of developing CIE.

While ischemic stroke is a well-established risk factor for CIE due to chronic BBB disruption, the potential role of prior intracerebral hemorrhage remains speculative. There is currently no published evidence directly linking a history of remote intracerebral hemorrhage to increased risk of CIE. To our knowledge, this is the first reported case. This suggests that remote hemorrhagic brain injury may represent an underrecognized risk factor, warranting further investigation in vulnerable populations.

## Case presentation

A 64-year-old woman with a medical history of ESRD on thrice-weekly hemodialysis, hypertension, type 2 diabetes mellitus, coronary artery disease, and hypothyroidism presented to the emergency department with a decreased level of consciousness. Her neurological history included a remote right temporoparietal lobar hemorrhage in 2021 and a recent left thalamic hemorrhage diagnosed one week prior to this admission of 2025. At that time, she had presented with acute right-sided weakness. Neuroimaging revealed a left thalamic hematoma, and she was admitted for monitoring. After stabilization, she was transferred to inpatient rehabilitation for recovery. During rehabilitation, she remained alert, with stable residual right hemiparesis and no cognitive deficits until the day of her current presentation.

The day prior to this admission, she underwent routine hemodialysis. That evening, she experienced insomnia and was given multiple medications, including gabapentin, quetiapine, ramelteon, melatonin, and hydrocodone. Afterward, she became progressively less responsive. The following morning, she was found to be minimally verbal and unable to participate in rehabilitation activities, though her motor strength remained unchanged. She was transferred to the hospital for further evaluation.

**Figure 1:**
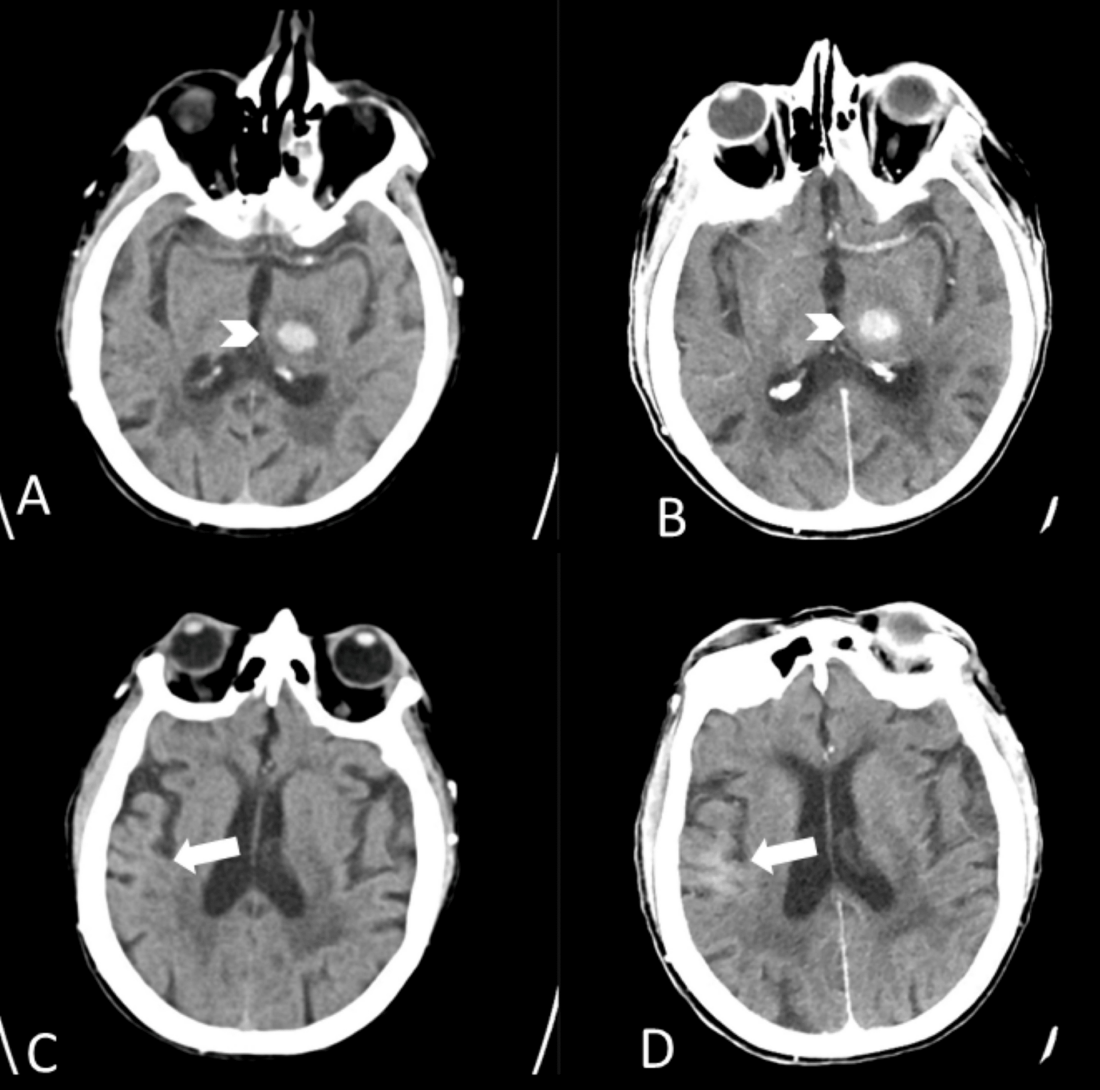
CT images at admission and follow-up non-contrast head CT obtained six hours after CTA of the head and neck A, C: initial CT shows an isolated left thalamic hemorrhage (arrowhead) without any hyperdensity in the right temporoparietal region (arrow); B, D: follow-up CT demonstrates a stable left thalamic hemorrhage (arrowhead) and a new diffuse hyperdensity (arrow) in the right temporoparietal lobe, located superior to the right insula.

In the emergency department, her vital signs were stable. Neurologically, she was awake but minimally verbal, able to state her name and follow simple commands. There were no new focal deficits; her pre-existing right-sided spastic hemiparesis was unchanged. Initial laboratory evaluation was unremarkable aside from a mildly elevated creatinine, consistent with her ESRD baseline (Table 1). Non-contrast head CT demonstrated a stable left thalamic hematoma measuring 15x13x15 mm with mild perilesional edema and a 2 mm rightward midline shift (Figure 1). Due to concern for possible acute stroke, a CT angiography (CTA) of the head and neck was performed with 80 mL of Omnipaque 350 (total iodine dose 28 grams), revealing no evidence of large vessel occlusion (not shown).

**Table 1:** Laboratory results from initial presentation Laboratory tests were unremarkable aside from a mildly elevated creatinine, with no significant electrolyte abnormalities. WBC: white blood cell; RBC: red blood cell; HGB: hemoglobin; PLT: platelet; BUN: blood urea nitrogen

Blood/Serum	Admission	Reference
WBC	5.53	4.5-11 x103/uL
RBC	4.07	3.5-5.5 x106/uL
HGB	13.1	12-15 g/DL
PLT	133	150-450 x103/uL
BUN	21.1	7-17 mg/dL
Creatinine	4.27	0.52-1.04 mg/dL
Sodium	132	136-145 mmol/L
Potassium	4.7	3.3-4.7 mmol/L
Glucose	191	74-106 mg/dL
Anion Gap	19	5-19 mmol/L

She was admitted to the regular floor with a diagnosis of evolving left thalamic hemorrhage and presumed encephalopathy due to polypharmacy. Approximately six hours after contrast administration, her mental status acutely worsened. She became obtunded, nonverbal, and unresponsive to verbal stimuli, though she retained spontaneous movement of all four extremities. An emergent repeat head CT showed stable left thalamic hemorrhage but new hyperdensity in the right temporoparietal region consistent with contrast extravasation at the site of prior lobar hemorrhage (Figure 1).

**Figure 2:**
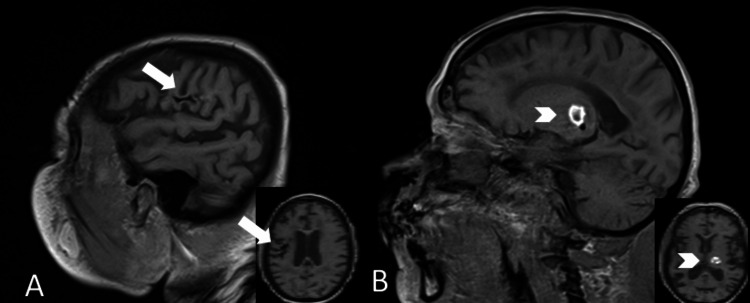
Comparison of T1-weighted MRI signal characteristics of the two lesions on sagittal views with corresponding axial views shown in small windows A: the right temporoparietal lesion (arrow) shows linear subcortical T1 hypointensity consistent with remote hemorrhage, without evidence of new hemorrhage; B: in contrast, the left thalamic lesion (arrowhead) demonstrates focal T1 hyperintensity, consistent with subacute hemorrhage.

**Figure 3:**
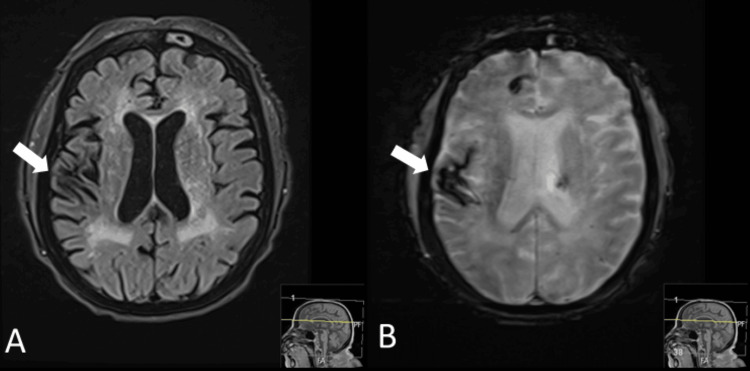
Corresponding MRI findings of the right temporoparietal lobe lesion on T2-weighted and SWI sequences A: linear T2 hypointensity in the right temporoparietal region consistent with encephalomalacia (arrow); B: susceptibility artifact in the same region on SWI indicates chronic hemosiderin deposition (arrow), consistent with prior hemorrhage. SWI: susceptibility-weighted imaging

She was transferred to the neurocritical care unit. Stat EEG revealed moderate diffuse encephalopathy with mixed-frequency slowing, triphasic waveforms, and multifocal epileptiform discharges, which were suggestive of a toxic/metabolic encephalopathy with seizure potential, but without any seizures. On hospital day 2, she was started on renal-adjusted levetiracetam and resumed hemodialysis. An MRI of the brain confirmed a stable left thalamic hemorrhage and remote right temporoparietal encephalomalacia without evidence of acute infarct or new hemorrhage (Figures 2, 3). The MRI findings further support that the new hyperdensity in the right temporoparietal lobe observed on the second CT after contrast administration represents contrast extravasation rather than a new hemorrhage.

Over the following week, her mental status gradually improved with regular dialysis. By hospital day 7, she was alert, able to state her and her son’s names, and followed commands consistently. However, she failed a swallowing evaluation and underwent percutaneous gastrostomy tube placement. She was ultimately transferred back to inpatient rehabilitation for continued recovery.

## Discussion

This case highlights several important and novel aspects of CIE. First, the development of CIE after a relatively low dose of contrast, 80 mL of Omnipaque 350, corresponding to approximately 28 grams of iodine, is unusual. Most reported cases of CIE involve contrast doses exceeding 100 mL, particularly during angiographic procedures [1]. This suggests that the patient had additional risk factors predisposing her to CIE. Second, her neurological decline occurred six hours after contrast administration rather than immediately, supporting the hypothesis that, in the setting of ESRD, contrast may persist in the circulation and gradually accumulate in the brain. Third and most importantly, imaging revealed contrast extravasation into the site of a remote cerebral hemorrhage, supporting the hypothesis that a chronically disrupted BBB from prior hemorrhage can serve as a significant risk factor for CIE, particularly in patients with impaired renal clearance.

The prior right temporoparietal lobar hemorrhage in this patient had occurred four years earlier, yet imaging showed new contrast hyperdensity specifically localized to this region. This finding is consistent with contrast pooling in an area of chronic encephalomalacia. BBB disruption is well-documented in intracerebral hemorrhage [6,7]; such dysfunction could persist long after the initial insult. Chronic encephalomalacic regions may retain structural and microvascular abnormalities that impair BBB integrity. These chronic changes could render the affected tissue more permeable to exogenous substances such as iodinated contrast agents. Thus, even years after the hemorrhagic event, regions of prior intracerebral hemorrhage may serve as reservoirs for delayed contrast extravasation, particularly in vulnerable patients with additional risk factors such as ESRD, which impairs contrast clearance. This case illustrates that remote hemorrhagic sites should be recognized as potential loci of contrast leakage and neurotoxicity due to chronically compromised BBB function.

In addition to prior cerebral hemorrhage, the patient's ESRD likely played a critical role in predisposing her to CIE. Normally, iodinated contrast agents are renally excreted within 24 hours. In patients with ESRD, contrast can remain in circulation for days without dialysis, significantly prolonging brain exposure. Though the efficacy of dialysis in reversing CIE is debated, some studies suggest that prompt dialysis after contrast exposure may reduce systemic toxicity and facilitate neurologic recovery [4,8]. In our case, the patient’s gradual improvement following resumed hemodialysis supports this notion.

The diagnosis of CIE is often one of exclusion. In this case, a comprehensive evaluation ruled out acute ischemic stroke, new intracranial hemorrhage, infection, or metabolic derangements aside from her baseline renal dysfunction. Although the patient had ESRD and underwent dialysis just one day prior with creatinine at her baseline, her neurological decline was not attributable to these factors, supporting the diagnosis of CIE. There is no standardized treatment for CIE, and management remains largely supportive, involving close neurological monitoring, hemodynamic stabilization, and ensuring adequate hydration to facilitate contrast clearance [9]. In patients with ESRD, prompt initiation or continuation of dialysis may help accelerate the removal of contrast agents from circulation, although evidence on its efficacy in preventing or mitigating CIE is limited [8]. Corticosteroids and antiepileptic drugs have been used in some cases, particularly when cerebral edema or seizures are present, but their benefit is anecdotal [9]. In this case, we initiated levetiracetam in response to EEG findings suggestive of toxic or metabolic encephalopathy with a possible seizure component. Steroids were not administered, as neuroimaging showed no evidence of cerebral edema. Most patients with CIE recover fully within 48 to 72 hours. However, severe or prolonged cases, particularly in individuals with multiple risk factors, as in our patient, may require intensive supportive care and longer recovery times.

From a radiologic standpoint, CIE can be mistaken for a new intracranial hemorrhage on CT due to hyperdense contrast accumulation. Contrast extravasation may be misinterpreted as either intraparenchymal hemorrhage or subarachnoid hemorrhage (SAH). For example, in our case, the CT was interpreted by the radiologist as SAH. Differentiating contrast from blood on CT can be challenging, particularly in patients with complex neurological histories. In this case, the hyperdensity was localized to an area of chronic encephalomalacia and lacked associated mass effect or surrounding edema findings more consistent with contrast extravasation than with acute bleeding. MRI further supported the diagnosis by confirming the absence of new hemorrhage or infarction. Another helpful imaging modality is dual-energy CT, which can distinguish iodine-based contrast from blood by analyzing attenuation differences at varying energy levels. Contrast shows stronger attenuation at low energies and less at high energies, whereas blood shows less variability [10]. However, dual-energy CT may not be available in all clinical settings.

This case underscores the importance of clinician vigilance when administering contrast to high-risk populations, particularly patients with ESRD and a history of central nervous system (CNS) injury. Currently, there are no established guidelines for contrast administration in patients with prior intracerebral hemorrhage or stroke. However, our findings suggest that such individuals may benefit from individualized risk assessment, avoidance of unnecessary contrast exposure, or use of reduced contrast volumes when imaging is essential. Notably, to our knowledge, this is the first documented case of CIE associated with contrast leakage into a site of remote cerebral hemorrhage. While additional data is needed, this observation suggests that areas of prior hemorrhage, especially in patients with impaired renal clearance, may represent high-risk zones for CIE. Increased awareness of this potential complication may inform future clinical decision-making and imaging protocols. Further study is warranted.

## Conclusions

This case highlights a rare presentation of CIE due to contrast extravasation at a site of remote cerebral hemorrhage in a patient with ESRD. It raises the possibility that prior CNS insults may predispose to localized BBB vulnerability, allowing contrast to penetrate previously damaged regions of the brain. While CIE is typically associated with large contrast volumes, this case suggests that even low-dose contrast may precipitate encephalopathy in high-risk individuals. Clinicians may need to exercise heightened caution when administering contrast to patients with ESRD with a history of intracranial pathology and consider early dialysis and supportive care in cases of suspected CIE.
